# Addressing Women's Non-Maternal Healthcare Financing in Developing Countries: What Can We Learn from the Experiences of Rural Indian Women?

**DOI:** 10.1371/journal.pone.0029936

**Published:** 2012-01-17

**Authors:** Saji S. Gopalan, Varatharajan Durairaj

**Affiliations:** 1 Blair East, Silver Spring, Maryland, United States of America; 2 Department of Health Systems Financing, World Health Organization, Geneva, Switzerland; Yale University School of Medicine, United States of America

## Abstract

**Background and Objectives:**

This paper focuses on the inadequate attention on women's non-maternal healthcare in low- and middle-income countries. The study assessed the purchase of and financial access to non-maternal healthcare. It also scoped for mainstreaming household financial resources in this regard to suggest for alternatives.

**Methods:**

A household survey through multi-stage stratified sampling in the state of Orissa interviewed rural women above 15 years who were neither pregnant nor had any pregnancy-related outcome six weeks preceding the survey. The questions explored on the processes, determinants and outcomes of health seeking for non-maternal ailments. The outcome measures were healthcare access, cost of care and financial access. The independent variables for bivariate and multivariate analyses were contextual factors, health seeking and financing pattern.

**Results:**

The survey obtained a response rate of 98.64% and among 800 women, 43.8% had no schooling and 51% were above 60 years. Each woman reported at least one episode of non-maternal ailment; financial constraints prevented 68% from receiving timely and complete care. Distress coping measures (e.g. borrowings) dominated the financing source (67.9%) followed by community–based measures (32.1%). Only 6% had financial risk-protection; financial risk of not obtaining care doubled for women aged over 60 years (OR 2.00, 95% CI 0.84–4.80), seeking outpatient consultation (OR 2.01, 95% CI 0.89–4.81), facing unfavourable household response (OR 2.04, 95% CI 1.09–3.83), and lacking other financial alternatives (OR 2.13, 95% CI 1.11–4.07). When it comes to timely mobilization of funds and healthcare seeking, 90% (714) of the households preferred maternal care to non-maternal healthcare.

**Conclusion:**

The existing financing options enable sub-optimal purchase of women's non-maternal healthcare. Though dominant, household economy extends inadequate attention in this regard owing to its unfavourable approach towards non-maternal healthcare and limited financial capacity and support from other financial resources.

## Introduction

Though investing on maternal health alone is not sufficient for a healthy future, globally, maternal health has almost become synonymous with women's health in policy circles [1]–[2]. A closer look at priorities of ‘Millennium Declaration’ on women also indicates the dominance of maternal health [3]. India demonstrates a divergence in this regard as health policies focus largely on women's essential healthcare needs, while the consequent women-centerd health programs and initiatives are practically confined to maternal health [4].The flagship program, National Rural Health Mission (NRHM) with ‘Reproductive and Child Health’ as its pivot is a fine example in this regard [5]. Many LMICs experienced grooming of healthcare facilities, human resources, financial incentives, evaluation frameworks, community-based programs and inter-sectoral convergence geared for maternal and child health [4].

Policy negligence leads to gradual sup-optimal resource base for women's non-maternal healthcare in LMICs including India [6]. Such countries meet with deaths of millions of women from preventable and treatable illnesses (e.g. lower respiratory infections, diarrheal diseases etc.) yearly [6]. Women require more resources to tackle non-maternal healthcare needs compared to men, as they have higher life expectancy and prevalence of illnesses like non-communicable chronic diseases [6]. In India, non-maternal healthcare constitute over 50% of women's healthcare needs, yet it receives only 25% of the government spending on women's healthcare [7].

The shift of incidence of non-maternal healthcare expenditure on households from the government is not expected to be for its favour as women face unfavourable gender power structure in households [8]–[9]. Further, the consideration of child birth as a ‘family event’ might pose limited resource availability for non-maternal health among diversified household healthcare needs [10]. In this context, the study assessed the purchase of and financial access to women's non-maternal healthcare. It also assessed the scope for mainstreaming household financial resources for non-maternal healthcare to suggest alternative ways in this regard. The financing options explored were household economy, government and alternative sources. The study outcomes are expected to enrich the existing limited evidence base on financing for women's non-maternal healthcare.

### Conceptualizing non-maternal healthcare financing

**Figure 1:**
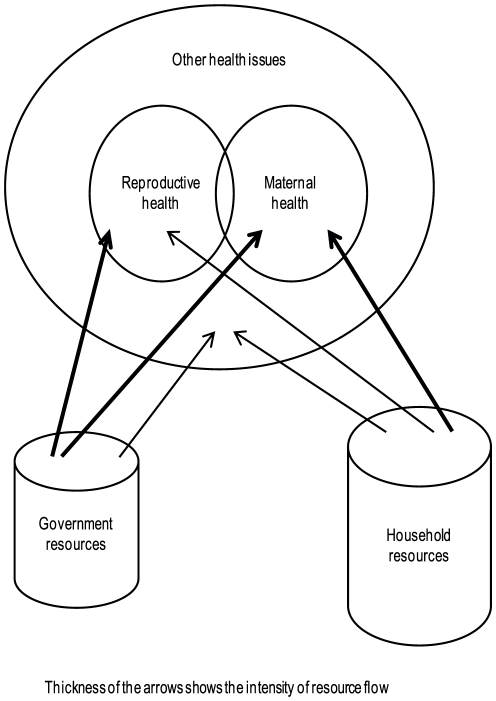
Existing healthcare financing context for women in low- and middle-income countries.

Addressing women's healthcare needs is complex, as they have two sets of requirements namely, maternal and non-maternal [10]. Non-maternal ailments like ischaemic heart disease, tuberculosis, injury, cancer etc. account for nearly a half deaths among women aged between 20 and 59 years in LMICs [1]. This could be attributed to less than optimal resource allocation for non-maternal healthcare by government and non-government financing sources compared to maternal healthcare (Figure-1). To overcome this asymmetry in resourcing, an understanding of the existing financial barriers for non-maternal healthcare and their extent is essential. We considered any care required for any ailment of women other than that related to the conditions of pregnancy, child birth and contraception (recognised more as a device for birth control than a healthcare requirement) as part of non-maternal healthcare. We defined a financing source for non-maternal healthcare as any financial means (e.g. savings, insurance, micro credits, government transfer, borrowing, sale of assets etc.) which enables the purchase of healthcare.

## Materials and Methods

### Study setting

It was a population-based cross-sectional study in *Angul* and *Malkangiri* districts of *Orissa* [11]–[13]. *Angul* is centrally located with relatively better development indicators compared to *Malkangiri*. *Angul* has a Human Development Index of 0.66 (*Malkangiri* 0.57,*Orissa* 0.37); Gender Development Index at 0.63 (*Malkangiri* 0.41, *Orissa* 0.55); female literacy of 54% (*Malkangiri* 48%, *Orissa* 52.0%), and 102,076 rural households (*Malkangiri* 196,825).

### Ethics statement

The study was approved by the academic research committee of Kerala University. Study objectives were clearly explained to each woman along with the use of information she would be revealing. Based on this clarification, their written informed consent was obtained (witnessed thumb impression for non-literates) from each of them. Participation in the study was voluntary and the women had the options to refuse answering any question and withdraw at any point of time of the interview. The questions were asked in a culturally appropriate manner and the interviews were conducted at a locally convenient time with the community consensus. Identities of the respondents were removed and kept confidential during the data entry and subsequent analysis.

### Survey design

Through a multi-stage random stratified sampling, we selected 800 women, above 15 years who were neither pregnant nor had any pregnancy-related outcome six weeks preceding the survey. The first stage selected Orissa among developing Indian states, followed by the districts of *Angul* and *Malkangiri* (each from the top- and bottom-five districts of human development ranking, 2004), and 50% of rural administrative divisions from each district. Finally, households with at least one eligible woman were randomly selected after ‘line-listing’. The selected blocks together had 117,142 households (about 500,000 population) and 8,112 (38%) of them were line-listed as eligible. Targeting a 10% of the eligible households, we could survey only 800 women (i.e. one woman per household) at a response rate of 98.64%.The locally based women's groups provided support on identifying eligible households and familiarity with the respondents before the survey.

The household survey conducted during August-September 2008, used a structured and pre-tested interview schedule (in local language, *Odia*). There were both open and close ended questions, seeking information on purchase of women's non-maternal healthcare for the last episode of illness during six weeks preceding the survey. The survey explored; processes of health seeking (e.g. timing of care seeking and provider), determinants of care seeking (i.e. contextual factors) and outcomes of care seeking (timely and complete care and financial catastrophe), given in appendix S1. ‘Contextual factors’ constituted the background information of women (socio-economic and demographic), financing context (type, pattern of resource mobilization, perceived financial constraints) and health related factors (type of illness, perceived seriousness of illness and nature of care required i.e. inpatient/outpatient). Wherever feasible, we validated the information given by the women with supporting evidences such as prescriptions, hospital and pharmacy bills.

### Outcomes of interest and Independent variables

There were three outcome variables namely, healthcare access, cost of care and financial access. ‘Gaps in access to non-maternal healthcare’ for the most recent episode were captured in three dimensions - non-treatment, delayed treatment (over 2 days from the onset), and incomplete treatment (discontinuing care before the illness is completely cured). As Orissa is prone to infectious diseases like malaria, we referred to similar studies in Orissa to select the time period for calculating delay in treatment [14]. ‘Financial access’ demonstrated the availability of financial resources to access care within two days of the onset of illness and continue the care till it gets completely cured. ‘Treatment cost’ captured the total cost of each woman on drugs, consultation, diagnosis, surgery, transportation, hospitalization and escort. We selected these outcome variables based on the existing evidences on the determinants and products of a healthcare purchase [1]. The common predictor variables were ‘contextual factors’ (including financing pattern) and ‘health seeking pattern’.

### Statistical analysis

Apart from univariate analyses on contextual factors, certain bivariate and multivariate analyses were conducted to obtain the association of various predictors on the determinants and outcomes of the purchase of non-maternal healthcare during the reference period. The study design pre-specified certain bivariate analyses to understand the association of independent variables with; 1) gaps in access to non-maternal healthcare, 2) reported financial access to timely and complete non-maternal healthcare, and 3) cost of non-maternal healthcare. The multivariate test was pre-built in the study design to derive the predictors of the outcome variables, depending on the associations drawn from bivariate analyses of pre-specified predictors. Microsoft Excel was used for data entry and SPSS version 17.0 was used to analyse the data.

## Results

### Background characteristics of women

Among the 800 women surveyed, 90.5% (n = 724) were from socially backward classes, 69.6% (n = 557) lived in poor quality houses, and 64.5% (n = 516) were below the poverty line, 43.8% (n = 350) had no schooling, 51% (n = 406) were above 60 years, 41% (n = 329) did not earn any income, 62% (n = 498) did not involve in any banking activities, 76% (n = 610) were engaged in microfinance activities, and 74.5% (n = 596) did not have financial autonomy (Table 1&2).

**Table 1:** Characteristics of households (N = 800).

Characteristics	No. of households (%)
***House type***	
*Pucca* (Concrete/tiled roof, wall and cement floor)	243 (30.4)
*Kachcha* (Thatched roof, mud floor and wall)	557 (69.6)
***Social Classes***	
Scheduled tribe*	164 (20.5)
Scheduled caste*	70 (8.8)
Other Backward Community	490 (61.2)
Others	76 (9.5)
***Level of poverty***	
Below poverty line#	516 (64.5)
Above poverty line	284 (35.5)
***Family Type***	
Joint	532 (66.5)
Nuclear	268 (33.5)

*Scheduled tribe and scheduled caste are considered as socioeconomically marginalized populations and receive special focus and privileges from the Federal Government.

# *Below poverty line households are those living on <$1 per capita/day as per current Indian estimation. In our survey a household was listed as ‘below poverty line’ if it possessed the social security identification card issued by the Federal Government indicating its poverty status, not based on income reported by respondents.*

**Table 2:** Background characteristics of women (N = 800).

Characteristics	No. of women (%)
***Age (in years)***	
18–30	118 (14.8)
31–45	63 (8.0)
46–60	213 (26.2)
>60	406 (51.0)
Median (range)	42 (18–67)
***Years of schooling***	
0	350 (43.8)
01–05	312 (39.0)
06–10	62 (7.7)
>10	76 (9.5)
Median (range)	6 (0–12)
***Occupation***	
Homemakers@	329 (41.1)
Daily-wage labourer	234 (29.3)
Self-employed	194 (24.3)
Employed in government	21 (2.6)
Employed in private	22 (2.8)
***Monthly individual income (US$)***	
0	332 (41.3)
<10	384 (48.0)
10–20	78 (9.6)
>21	6 (0.6)
Median (range)	8 (0–40)

@ *Home makers are women who are not productively employed and do not earn any income.*

### Non-maternal healthcare needs, demand and gap

Every woman met with at least one episode of non-maternal ailment during the reference period of six weeks. They were affected by malaria (22.3%, n = 178), reproductive tract infection (14.8%,n = 118), asthma (12.8%, n = 102), fever (12.5%, n = 100), typhoid (11.2%, n = 90), diarrhoea/dysentery (8.8%, n = 70), tuberculosis (7.7%, n = 62), body/back/head ache (6%, n = 48) and skin/ear/eye/tooth diseases (4%, n = 32). The median duration of illness was four days; 65.3% women (n = 526) had illness for less than a week, and 3.3% women (n = 26) were sick for more than a month (Table-3).

**Table 3:** Particulars of non-maternal ailments, care seeking and determinants.

Characteristics	No. of women (%)
***Non-maternal ailments***	
Malaria	178 (22.3)
Reproductive tract infection	118 (14.8)
Asthma	102 (12.8)
Fever	100 (12.5)
Typhoid	90 (11.2)
Diarrhoea/dysentery	70 (8.8)
Tuberculosis	62 (7.7)
Body/back/head ache	48 (6.0)
Skin/ear/eye/tooth diseases	32 (4.0)
*Total*	*800(100)*
***Healthcare seeking***	
Received care	360 (45.0)
Not received care	440 (55.0)
*Total*	*800(100)*
***Reasons for non-seeking care***	
Financial limitations	284 (64.3)
Perceived non-seriousness	125 (28.3)
Residing far from health centers	31 (7.4)
*Total*	*440(100)*
***Presence of financial limitations for timely and complete care***	
Yes	526 (88)
No	72(12)
*Total*	*598(100)*
***Household response to non-maternal healthcare***	
Preferred maternal care over non-maternal healthcare	714 (89.2)
Equal weight to both maternal and non-maternal healthcare	86 (10.8)
*Total*	*800(100)*

### Financial access to non-maternal healthcare

Only 45% women (n = 360) sought care during illnesses, while only 32.5% (n = 260) had timely care and 25.2% (n = 202) had both timely and complete care (or did not have any gaps in access to care). Among those who did not seek care, financial limitations restricted 64.5% (n = 284), perceived non-seriousness and residing far from health centers affected 28.4% (n = 125) and 7.0% (n = 31) respectively (Table-3). Within the group of those who sought care, financial limitations restricted 27.7% (n = 98) from receiving timely care and another 23.3% (n = 84) complete care.

Amidst the various predictors of ‘gaps in access to non-maternal healthcare’, the association of reported financial constraints is found out to be statistically significant (chi-square P<0.05). We further decategorized ‘gaps in access to non-maternal healthcare’ into ‘timely and complete care’ and explored their association with reported financial constraints. In the category of those who reported financial constraints, only 10.8% received timely and complete care; whereas it was 83.1% among women who did not report financial constraints. Being aged over 60 years doubled the chance of reported financial constraints; similar odds ratios were found among those who reported unfavourable household response towards non-maternal healthcare, lacked financial alternatives and required outpatient care (Table-4).

**Table 4:** Predictors of reported financial access to timely and complete non-maternal healthcare.

Predictors	Adjusted Odds Ratio#	95% CI	P Value
**Age**			
>60 years*	1	-	
≤60 years	**2**	0.84–4.80	**0.01**
**Household response**			
Unfavourable household response*	1	-	
Favourable household response	**2.04**	1.09–3.83	**0.03**
**Alternative financing sources**			
Absence of alternative financing sources	1	-	
Presence of alternative financing sources*	**2.13**	1.11–4.07	**0.02**
**Nature of care required**			
Outpatient*	1	-	
Inpatient	**2.01**	0.89–4.81	**0.01**

*Reference category in the multiple logistic regression.

# *- Adjusted for social classes, house type, family type, poverty status, personal income, and educational status.*

### Cost of non-maternal care

For those women (45%, n = 360) who sought treatment for non-maternal healthcare, the median treatment cost for the last episode was US$ 24. Decomposition of cost further yields the relative contributions of different health service categories; surgery contributed 32.2%, followed by medicines (31.6%), consultation (8.3%), transportation (8.3%), spending on escorts (8.3%), hospital stay (7.1%) and diagnosis (4.2%).

#### Cost determinants

We explored the association between predictor variables (derived from bi-variate analysis whose chi-square P<0.05) such as nature of illnesses, social community, age, delays in treatment, provider, type of alternative financing source and cost of care (Table-5). Women who were socially backward (2.12 times), delayed availing care for more than a week (2.01 times), and aged above 60 years (2.03 times) had more likelihood of incurring higher cost of care than their counterparts.

**Table 5:** Determinants of cost of care of non-maternal healthcare.

Predictor Variables	Adjusted Odds Ratio#	95% CI	P value
**Delay in treatment**			
>7days*	1	-	**-**
≤7days	**2.01**	0.89–4.46	**0.01**
**Social class**			
Backward classes*	1	-	**-**
Forward classes	**2.12**	1.09–3.70	**0.03**
**Age**			
>60 years*	1	-	-
≤60 years	**2.03**	0.93–4.39	**0.02**

*Reference group in the multiple logistic regression.

# *- Adjusted for house type, family type, poverty status, personal income, and educational status.*

### Household response to women's health care needs

We assessed the relative differences in the household approach towards maternal and non-maternal healthcare. The differential approach was captured in terms of their preferences for timely healthcare seeking (i.e. within two days from the onset of illness) and timely mobilization of funds to enable purchase of care. About 90% (n = 714/800) of the households preferred maternal care over non-maternal healthcare for timely mobilization of funds and care seeking, while 10.8% (n = 86) paid equal attention to both (Table-3). The pattern was almost uniformly spread across different socioeconomic groups, except for different age groups (i.e. between >60 and ≤60 age groups) as observed from the chi-square test (P<0.05). There was no significant association between the favourable household response and presence of financial protection measures (chi-square P = 0.26). Most of the households (52.5%, n = 420/800) were reported to have better knowledge of women's pregnancy care needs compared to that of non-maternal healthcare. While, 47.6% (n = 381/800) were reportedly having more concern on women's pregnancy care than non-maternal healthcare as the former was perceived to be a matter of child birth than something related to women's health.

### Household financing sources for non-maternal healthcare

Majority of the women used multiple sources of financing for the last episode of non-maternal healthcare. Most of the women (59.7%, n = 215/360) depended on loans from different formal and informal sources, 32.2% (n = 116) approached community-based financing measures and 8.1% (n = 29) sold off their assets to mobilize funds. The sources of loans were formal banks with or without mortgage (34.8%, n = 75/215), micro credits (34.4%, n = 74), and high-interest informal loan from unorganised money lenders (30.7%, n = 66). The community-based financing measures included health insurance schemes for inpatient care (45.7%, n = 53/116) and micro-finance institution linked revolving funds for outpatient care (54%, n = 63/116), especially for drugs.

Though 30% (n = 109/360) of the women received free drugs and consultations through some of the government schemes (e.g. malaria, diarrhoea etc.), they depended on the above sources to meet the additional expenses (diagnosis, accommodation etc.). Though many used community based pre-payment measures, due to an absence of a comprehensive financial risk-protection coverage, 99% (n = 115/116) of them had to incur some on-the-spot expenses. Majority (65.7%, n = 50/74) of the women who used micro credits reported to use them for emergencies like accidents and surgery.

Although 26.3% (n = 210/800) households had insurance protection, only half of them (50%, n = 105) could provide any kind of coverage to the surveyed women. This less coverage of women was due to the exclusion of elderly women as insurance schemes largely avoided extended family members or they covered only four household members. Among them, only half (50%, n = 53/105) could avail the benefits for the last episode of non-maternal healthcare. The non-users group of health insurance schemes consisted of women who did not require in-patient care (34%, n = 18/53), did not want to exhaust the ‘sum assured’ (34%, n = 18/53), and had exhausted the ‘sum assured’ (32%, n = 17/53). In short, 93.4% (n = 747/800) of the women did not have any financial risk-protection measure for the last episode of non-maternal healthcare due to lack of money, limited family support and non-comprehensive nature of insurance mechanisms to protect non-maternal healthcare.

#### Who mobilized household resources (on-the-spot payment or pre-pooling)?

Spouses took the responsibility to mobilize funds for non-maternal healthcare for 71.4% (n = 257/360)women; while for others, spouse's family members (10%, n = 37), own children and parents (9.5%, n = 35), women themselves (5.3%, n = 19), jointly with their spouse (3%, n = 12) mobilized funds. This mobilization of resources, especially through pre-payment options was not meant exclusively for non-maternal healthcare, but included other household healthcare needs.

#### Timeliness of financing

Among those who received care, 34%, n = 122/360 (32.1% with community-based pre-pooling measures) had already mobilized money when they faced illness; 14.5% (n = 52) had to mobilize it at the time of illness; it took a week for15.4% (n = 55), and the rest 36.1% (n = 130) required more than a week.

## Discussion

This paper is one of the first attempts in developing health systems to analyse the consumption of non-maternal healthcare and bring out evidences to streamline household financing in this regard. Though the study is confined to rural settings, the policy and household level prioritizations for maternal care do exist in urban settings, especially in the Asian context [15].

The study outcomes exhibit a clear household prioritization, irrespective of socioeconomic status, in favour of maternal care despite women having considerable non-maternal healthcare needs. Women underwent further adversities as most of the non-maternal healthcare needs are not adequately programmed into the health financing mechanisms [16].Consequently, they faced sub-optimal purchase of non-maternal healthcare with expected long term adverse health and socio-economic effects [17].

Financial risk-protection for non-maternal healthcare was provided by four main sources in the study setting such as formal insurance, free healthcare by government, community health insurance and microfinance. Though one-fourth of the sample households had cashless health insurance coverage for inpatient care, only a few could make use of them. The major constraints were their unorganized nature, limited resource pooling and low household prioritization for non-maternal healthcare [18].

However, other community-based measures linked with micro-finance institutions helped women for outpatient care particulalry to access drugs for mild illnesses. As most of the non-maternal healthcare needs require outpatient consultations, insurance schemes not covering outpatient care may not be very relevant [19]–[20]. Since outpatient care is a low-spending high-probable case, insurance may not be an attractive option as the premium will be unaffordable for many [21]. Gradually, women may postpone their treatment till the condition becomes worse enough to be eligible for insurance benefits. This scenario will push the cost of insurance up besides affecting the women's health as evident from study settings and elsewhere [22]. Similarly, if financial risk-protection measures combine both inpatient and outpatient care, there might be less utilization for non-maternal healthcare. This could be owing to their over-consciousness to keep ‘sum assured’ for emergency needs and household preference for other healthcare needs as seen in the study settings. Thus, in order to address women's non-maternal healthcare comprehensively, alternative non-insurance approaches could be developed catering to outpatient care. There are demonstrations of non-insurance schemes enhancing women's essential healthcare services in many low-income settings [23]. The government could offer supplementary funds to finance outpatient care and cater to the presently excluded extended family members like the elderly [24]–[25].

Among other financing sources, micro-credits were prominent, but were largely used to finance emergencies like accidents and surgery. Free healthcare provision by government also provided shield against financial catastrophe though not sufficiently. Distress coping measures like informal borrowings with high interest rate were predominant denoting the inevitability of comprehensive financial-risk protection for women's non-maternal healthcare [26]–[27].

Indian healthcare financing system is in transition and it aims at a predictable, accountable and sustainable healthcare financing framework like many other LMICs [22]. In addition to numerous community-based schemes, federal and state governments have also initiated new pre-payment health financing schemes [28]. While it is a welcome development, they should be able to identify and address comprehensively the non-maternal healthcare needs (both outpatient and inpatient). To fulfil it, we need an integrated financing approach to unify all the resources for effective pooling and utilization without duplication, besides achieving their intended purpose. Such an integrated approach could also enhance demand side awareness, which is a constraint as demonstrated by the investigated households.

**Figure 2:**
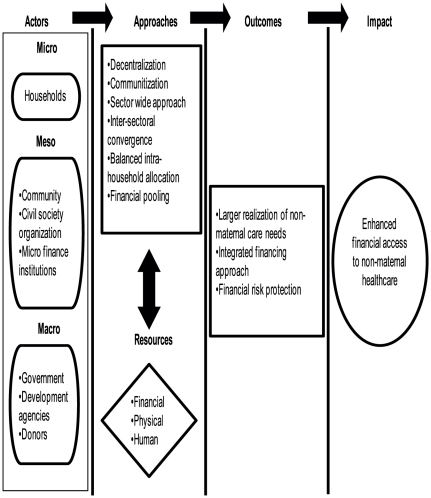
Trajectory of the proposed ‘integrated financing approach’ for non-maternal healthcare in LMICs.

Figure-2 proposes an option in this regard; confines its scope to build efforts to ensure financial access to non-maternal healthcare. This integrated approach may be applicable for many other LMICs with similar scenario. The framework provides role for each health financing actor. For instance, at the micro level, households need to ensure that intra-household resource allocation is optimised between maternal and non-maternal healthcare needs. At the meso level, it could be sensitization on non-maternal healthcare needs bringing in social, physical and financial resources for both risk-and non-risk pooling measures (e.g. equity funds for emergency non-maternal healthcare at the community and health center level). Ongoing efforts on decentralization and communitization of healthcare approach could be useful here [29]. The above measures invest on community resources and prefer discretionary resource allocation for essential needs and collective sensitization on health [30]–[31].At the macro level, enhanced financial provision and pooling for the comprehensive (out-patient and in-patient) health care including non-maternal healthcare is required. To ensure wider resource availability for non-maternal healthcare at macro level, we also need convergence with allied sectors on social protection measures. Such an integrated financial planning could be allied with service provision and empowerment of demand-side towards enhancing appropriate purchase of non-maternal healthcare.

### Conclusion

This paper highlights that women have substantial non-maternal ailments warranting significant financial attention. The current purchase of non-maternal healthcare is sub-optimal on behalf of household economy and other alternative financing options. Though household economy is the largest fund provider and many pre-payment alternatives existed, households could not adequately finance it. This indicates the households' limited ability to pre-pay for non-maternal healthcare, unfavourable approach of pre-payment options and households' less prioritization. Government strategies, schemes and financial incentives still largely favour maternal care and they are not coordinated and linked with other existing financing mechanisms. An integrated health financing framework with clearly defined roles for each mechanism and actor is necessary to streamline the resource flow and mainstream non-maternal healthcare. Community-based financing mechanisms could be used as a transitory channel to mainstream household resources towards non-maternal healthcare under a formal prepayment mechanism. Such a streamlining could further ensure the sustainability, predictability and accountability of those measures for non-maternal healthcare.

## Supporting Information

Appendix S1 **Household Survey Interview Schedule for women.**
(DOC)
