# Supplementary material for: Addressing Women's Non-Maternal Healthcare Financing in Developing Countries: What Can We Learn from the Experiences of Rural Indian Women?
Source: PLoS One. 2012 Jan 17;7(1):e29936. doi: 10.1371/journal.pone.0029936 (PMC3260165; doi:10.1371/journal.pone.0029936)
Supplement: Appendix S1 — Household Survey Interview Schedule for women. (DOC) [file pone.0029936.s001.doc]

**Appendix S1**

**Household Survey Schedule for interviewing women**

**Assessing the purchase and financial access to women’s non-maternal healthcare-Orissa, India**

Serial No**:**

|  |  |  |
| --- | --- | --- |

District: Village: Date of interview:

Interviewer name: Duration of interview: Respondent name:

| SECTION 1  BASIC INFORMATION ABOUT THE INDIVIDUAL |
| --- |

| NO. | QUESTIONS | RESPONSE | CODE |
| --- | --- | --- | --- |
| 101 | Age |  |  |
| 102 | Years of schooling |  |  |
| 103 | Occupation | (1) Housewife  (2) Casual labourer  (3) Govt employee  (4) Pvt. employee  (5) Self-employed  (6) Other----------------- |  |
| 104 | Monthly individual income (Rs) |  |  |
| 105 | Do you have bank account? | (1)Yes  (2)No |  |
| 106 | Are you involved in microfinance? | (1)Yes  (2)No |  |
| 107 | If no, what is the reason? |  |  |
| 108 | Do you have life insurance coverage? | (1)Yes  (2)No |  |
| 109 | Do you have any health insurance coverage? | (1)Yes  (2)No |  |
| 110 | Are you allowed to spend money as you wish | (1)Yes  (2)No |  |
| 111 | Who is making decisions on your health care? | (1)Yourself  (2)Husband/parent/child  (3)Jointly  (4)Other------------------- |  |
| 112 | Who is mobilising money for your health care? | (1)Yourself  (2) Husband/parent/child  (3)Jointly  (4)Other------------------- |  |

| SECTION 2  BASIC INFORMATION ABOUT THE HOUSEHOLD |
| --- |

|  | QUESTIONS | RESPONSE | CODE |
| --- | --- | --- | --- |
| 201 | Type of household | (1)Joint  (2)Nuclear |  |
| 202 | Type of house | (1) Pucca  (2)Kachcha |  |
| 203 | Nature of accommodation | (1)Own house  (2)Rented house  (3)Other---------------- |  |
| 204 | Social class | (1)SC  (2)ST  (3)OBC  (4)Other---------------- |  |
| 205 | Economic status | (1)APL  (2)BPL |  |
| 206 | Major occupation of head of household | (1)Govt.employee  (2) Pvt. employee  (3) Self-employed  (4) Casual laborer  (5) Other----------------- |  |
| 207 | Your relationship with head of the household | (1)Self  (2)Husband  (3) Parent  (4)In-law  (5) Child  (6) Other----------------- |  |
| 208 | Monthly household income |  |  |
| 209 | Number of males above 21years in the household |  |  |
| 210 | Number of females above 18 years in the household |  |  |
| 211 | Number of children in the household |  |  |
| 212 | Place of defecation | (1)Open  (2)Flush toilet within house  (3)Public/shared flush toilet  (4 )Pit toilet within house  (5) Public/shared pit toilet  (6) Other-------------------- |  |
| 213 | Source of drinking water | (1)Own piped water  (2)Hand pump  (3)Piped water (outside)  (4)Own well  (5) Public well  (6) River/canal/pond |  |
| 214 | No. of HH members other than you holding bank accounts |  |  |
| 215 | No. of HH members other than you involved in micro finance |  |  |
| 216 | No. of HH members apart from you holding  1.Life insurance policies  2.Health insurance policies |  |  |
| 217 | HH member responsible for household health care in terms of decision making |  |  |
| 218 | Who mobilizes resources for health care of the household |  |  |
| 219 | Household resource mobilization strategy for health care | (1) Past saving  (2)Bank loan  (3)Money lender  (4) Insurance (community/formal)  (5)Govt. transfer  (6) Microfinance  (7) Friends/relatives  (8) Selling assets  (9)Other--------------------- |  |
| 220 | No. of HH members other than you who availed health care during the last month three months? |  |  |
| 221 | Reason for seeking care by HH members? |  |  |
| 222 | Where do household members other than you go for treatment usually? | (1)Public doctor/facility  (2) Pvt. qualified doctor/facility  (3) Less than fully qualified practitioner(Ltfq)  (4) Pharmacy  (5) Other-------------------- |  |
| 223 | For what ailments do household members other than you seek care usually? |  |  |
| 224 | For what ailments do household members other than you NOT seek care usually? |  |  |
| 225 | Distance from nearest health provider | (1)0–5 KM  (2) 6–10 KM  (3) More than 10 KM |  |
| 226 | Distance form nearest government health centre e.g. Sub centre, PHC, CHC etc | (1)0–5 KM  (2) 6–10 KM  (3) More than 10 KM |  |
| 227 | How do household members other than you go to nearest health centre/ provider | (1)Walk  (2)Two wheeler  (3)Auto /Taxi  (4)Public Transport  (5) Other-------------------- |  |

| SECTION 3  HEALTH SEEKING BEHAVIOR FOR NON-MATERNAL CARE ( DURING LAST SIX WEEKS) [Please mention specifically on each episode] |
| --- |

| 301 | ***Illness and Treatment History*** | | | | | | | | | | | | | | | | | | |
| --- | --- | --- | --- | --- | --- | --- | --- | --- | --- | --- | --- | --- | --- | --- | --- | --- | --- | --- | --- |
|  | Illness | | | | | | Duration | | | Care sought from | | | | | | | | No. of times treatment sought | |
|  |  |  |  |  |  |  |  |  |  | Public | | | Pvt | | Ltfq | | Other |  |  |
|  | Body ache/head ache/common cold | | | | | |  | | |  | | |  | |  | |  |  | |
|  | Diarrhoea/vomiting | | | | | |  | | |  | | |  | |  | |  |  | |
|  | Leprosy | | | | | |  | | |  | | |  | |  | |  |  | |
|  | TB | | | | | |  | | |  | | |  | |  | |  |  | |
|  | Injury/accident | | | | | |  | | |  | | |  | |  | |  |  | |
|  | Malaria/filaria/Chikungunya | | | | | |  | | |  | | |  | |  | |  |  | |
|  | Eye/ear/skin/dental diseases | | | | | |  | | |  | | |  | |  | |  |  | |
|  | Reproductive health related problems | | | | | |  | | |  | | |  | |  | |  |  | |
|  | Respiratory infections | | | | | |  | | |  | | |  | |  | |  |  | |
|  | Diabetes | | | | | |  | | |  | | |  | |  | |  |  | |
|  | Blood pressure | | | | | |  | | |  | | |  | |  | |  |  | |
|  | Cardiac diseases | | | | | |  | | |  | | |  | |  | |  |  | |
|  | Other chronic diseases (specify) | | | | | |  | | |  | | |  | |  | |  |  | |
|  | Other------------------------- | | | | | |  | | |  | | |  | |  | |  |  | |
| 302 | Delays, non-treatment and incomplete treatment (for the above illness you suffered during last two weeks) | | | | | | | | | | | | | | | | | | |
|  | Illness | Delays (Days) | No treatment | | Incomplete treatment | | | | Reason for delay, no care or incomplete care | | | | | | | | | | |
|  |  |  |  |  |  |  |  |  | No money | | Too far | | | Not serious | | No permission | | | Other |
|  |  |  |  | |  | | | |  | |  | | |  | |  | | |  |
|  |  |  |  | |  | | | |  | |  | | |  | |  | | |  |
|  |  |  |  | |  | | | |  | |  | | |  | |  | | |  |
| 303 | Distance to provider and mode of transport (for the treatment of above illness) | | | | | | | | | | | | | | | | | | |
|  | Illness | Distance to provider(km) | | mode of transport | | | | | | | | | | | | | | | |
|  |  |  |  | Walk | | Two wheeler | | Bus/Public transport | | | | Auto/Taxi | | | | Ambulance | | | Other |
|  |  |  | |  | |  | |  | | | |  | | | |  | | |  |
|  |  |  | |  | |  | |  | | | |  | | | |  | | |  |
|  |  |  | |  | |  | |  | | | |  | | | |  | | |  |

| SECTION 4  **COST AND FINANCE (DURING LAST SIX WEEKS):** please mention specifically on each episode |
| --- |

| 401 | ***Cost of treatment (for the above illness)*** | | | | | | | | | | | | | | | | |
| --- | --- | --- | --- | --- | --- | --- | --- | --- | --- | --- | --- | --- | --- | --- | --- | --- | --- |
|  | Illness | Consultation | | Drugs | | Diagnostics | Transportation | | | Surgery | | Stay | | Escort/food | | | Total |
|  |  |  | |  | |  |  | | |  | |  | |  | | |  |
|  |  |  | |  | |  |  | | |  | |  | |  | | |  |
|  |  |  | |  | |  |  | | |  | |  | |  | | |  |
| 402 | ***Financing (for the above illness)*** | | | | | | | | | | | | | | | | |
|  | Illness | | Past savings | | Loan | | Friend/ Relative | | Insurance | | | | Mf | | | Govt. transfer | |
|  |  |  |  |  | Money lender | Bank |  |  | Formal | | CBHI | |  |  |  |  |  |
|  |  | |  | |  |  |  | |  | |  | |  | | |  | |
|  |  | |  | |  |  |  | |  | |  | |  | | |  | |
|  |  | |  | |  |  |  | |  | |  | |  | | |  | |
|  | QUESTIONS | | | | | | | RESPONSE | | | | | | | CODE | | |
| 403 | What was the mode of payment for the care? | | | | | | | (1)Cash on spot  (2)Cashless(voucher/card etc)  (3)Reimbursement  (4)Other-------------------------- | | | | | | |  | | |
| 404 | Who mobilized money for your health care? | | | | | | | 1)Yourself  (2) Husband/parent/child  (3)Jointly  (4)Other------------------------- | | | | | | |  | | |
| 405 | When was the money mobilized? | | | | | | | (1)Before the illness  (2) At the strike of illness  (3) Within two days  (4) 3-7 days  (5) More than 7 days | | | | | | |  | | |
| 406 | Did you have sufficient money in hand or at your disposal to pay | | | | | | | (1)Yes  (2)No | | | | | | |  | | |
| 407 | If no, how did you cope up? | | | | | | | ------------------------------------------------------------------------ | | | | | | |  | | |
| 408 | Did you face any difficulties in mobilizing money for care? If yes, please explain? | | | | | | | ------------------------------------------------------------------------ | | | | | | |  | | |
| 409 | Do you feel the above difficulties are different from those related to your maternal healthcare? If yes, how? | | | | | | | ------------------------------------------------------------------------ | | | | | | |  | | |
| 410 | Is there any difference in the way household responds to non-maternal related care compared to pregnancy care? | | | | | | | (1)Yes  (2)No | | | | | | |  | | |
| 411 | If yes, how different? | | | | | | | ------------------------------------ | | | | | | |  | | |
| 412 | Is there any difference in the household preference for timely care for maternal and non-maternal requirements | | | | | | |  | | | | | | |  | | |
| 413 | If yes, how different? | | | | | | | ------------------------------------------------------------------------ | | | | | | |  | | |
| 414 | Is there any difference in the household reference for timely resource mobilization between maternal and non-maternal healthcare? | | | | | | |  | | | | | | |  | | |
| 415 | If yes, how different? | | | | | | | ------------------------------------------------------------------------ | | | | | | |  | | |
| 416 | Is there any difference in the household members’ knowledge other than you on your maternal and non-maternal health care | | | | | | |  | | | | | | |  | | |
| 417 | If yes, how different? | | | | | | | ------------------------------------------------------------------------ | | | | | | |  | | |
| 418 | Even though your household is enrolled in insurance scheme, what is reason for not getting benefit from it for your last treatment? | | | | | | | ------------------------------------------------------------------------------------------------------------------------------------------- | | | | | | |  | | |
| 419 | Even though you are enrolled in insurance scheme, what is reason for not getting benefit from it for your last treatment? | | | | | | |  | | | | | | |  | | |
| 420 | What is the reason for you/household not having any insurance scheme? | | | | | | | ----------------------------------- | | | | | | |  | | |
| 421 | Do you think that it is necessary to have a financial risk-protection for non-maternal healthcare? Please mention why? | | | | | | |  | | | | | | |  | | |
| 422 | If any financing scheme is going to be introduced for non-maternal care, will you join? | | | | | | |  | | | | | | |  | | |
| 423 | If no, what is the reason? | | | | | | |  | | | | | | |  | | |
| 424 | If yes, how much you will be able to contribute per month (Rs)? | | | | | | |  | | | | | | |  | | |
| 425 | Do you think any external assistance will be required to pool the resources? Please mention why? | | | | | | |  | | | | | | |  | | |
